# First-dose ChAdOx1 and BNT162b2 COVID-19 vaccines and thrombocytopenic, thromboembolic and hemorrhagic events in Scotland

**DOI:** 10.1038/s41591-021-01408-4

**Published:** 2021-06-09

**Authors:** C. R. Simpson, T. Shi, E. Vasileiou, S. V. Katikireddi, S. Kerr, E. Moore, C. McCowan, U. Agrawal, S. A. Shah, L. D. Ritchie, J. Murray, J. Pan, D. T. Bradley, S. J. Stock, R. Wood, A. Chuter, J. Beggs, H. R. Stagg, M. Joy, R. S. M. Tsang, S. de Lusignan, R. Hobbs, R. A. Lyons, F. Torabi, S. Bedston, M. O’Leary, A. Akbari, J. McMenamin, C. Robertson, A. Sheikh

**Affiliations:** 1 School of Health, Wellington Faculty of Health, Victoria University of Wellington, Wellington, New Zealand; 2 Usher Institute, University of Edinburgh, Edinburgh, UK; 3 MRC/CSO Social & Public Health Sciences Unit, University of Glasgow, Glasgow, UK; 4 Public Health Scotland, Glasgow, Scotland; 5 School of Medicine, University of St. Andrews, St. Andrews, UK; 6 Centre of Academic Primary Care, University of Aberdeen, Aberdeen, UK; 7 Department of Mathematics and Statistics, University of Strathclyde, Glasgow, UK; 8 Queen’s University Belfast, Belfast, UK; 9 Public Health Agency, Belfast, Northern Ireland; 10 Health Data Research UK, BREATHE Hub, Edinburgh, UK; 11 Nuffield Department of Primary Care Health Sciences, University of Oxford, Oxford, UK; 12 Population Data Science, Swansea University, Swansea, UK

**Keywords:** Epidemiology, Epidemiology

## Abstract

Reports of ChAdOx1 vaccine–associated thrombocytopenia and vascular adverse events have led to some countries restricting its use. Using a national prospective cohort, we estimated associations between exposure to first-dose ChAdOx1 or BNT162b2 vaccination and hematological and vascular adverse events using a nested incident-matched case-control study and a confirmatory self-controlled case series (SCCS) analysis. An association was found between ChAdOx1 vaccination and idiopathic thrombocytopenic purpura (ITP) (0–27 d after vaccination; adjusted rate ratio (aRR) = 5.77, 95% confidence interval (CI), 2.41–13.83), with an estimated incidence of 1.13 (0.62–1.63) cases per 100,000 doses. An SCCS analysis confirmed that this was unlikely due to bias (RR = 1.98 (1.29–3.02)). There was also an increased risk for arterial thromboembolic events (aRR = 1.22, 1.12–1.34) 0–27 d after vaccination, with an SCCS RR of 0.97 (0.93–1.02). For hemorrhagic events 0–27 d after vaccination, the aRR was 1.48 (1.12–1.96), with an SCCS RR of 0.95 (0.82–1.11). A first dose of ChAdOx1 was found to be associated with small increased risks of ITP, with suggestive evidence of an increased risk of arterial thromboembolic and hemorrhagic events. The attenuation of effect found in the SCCS analysis means that there is the potential for overestimation of the reported results, which might indicate the presence of some residual confounding or confounding by indication. Public health authorities should inform their jurisdictions of these relatively small increased risks associated with ChAdOx1. No positive associations were seen between BNT162b2 and thrombocytopenic, thromboembolic and hemorrhagic events.

## Abstract (web summary)

New data from the EAVE II cohort in Scotland suggests that a first dose of the ChAdOx1 nCoV-19 vaccine might be associated with a small increase in the risk of idiopathic thrombocytopenic purpura between 0 and 27 d after vaccination.

## Main

The Coronavirus Disease 2019 (COVID-19) immunization program in the United Kingdom (UK) recommends COVID-19 vaccination for adults aged 18 and over. An independent UK-wide body, the Joint Committee on Vaccination and Immunisation (JCVI), has recommended vaccination of all adults beginning with those at highest risk of serious COVID-19 outcomes—in particular, hospitalizations and deaths (Supplementary Table 1)^1^. The three vaccines currently being administered in the UK—ChAdOx1 nCoV-19 (Oxford–AstraZeneca, hereafter ChAdOx1), BNT162b2 mRNA (Pfizer–BioNTech, hereafter BNT162b2) and mRNA-1273 (Moderna)—have been shown to reduce COVID-19 infections, hospitalizations and deaths^2–5^. Given that the first dose of mRNA-1273 was given in Scotland only on 7 April 2021, this article will focus on ChAdOx1 and BNT162b2.

The risk of adverse events after vaccine administration has been assessed in clinical trials. These found that the ChAdOx1 and BNT162b2 vaccines have been generally well tolerated^6,7^. The most commonly reported adverse events included injection site reactions, such as pain, redness, tenderness and swelling, and non-serious systemic reactions, such as myalgia, headache, nausea and fever^6–8^. Reports of serious adverse events have been rare^9^. However, concerns have been raised over the safety of the ChAdOx1 vaccine, and this has resulted in several countries initially temporarily suspending and then restricting use of ChAdOx1 to certain age groups^10–13^. By 4 April 2021, the European Medicines Agency (EMA) had received 169 reports of central venous thromboembolic events^14,15^, and an EMA signal assessment report on 8 April 2021 concluded that a signal of disproportionality was noted for rare events, such as disseminated intravascular coagulation, cerebral venous sinus thrombosis (CVST), as well as arterial thromboembolic and hemorrhagic stroke, which warranted further investigation^16^. The UK’s Medicines & Healthcare products and Regulatory Agency (MHRA) had received, as of 21 April 2021, 209 reports of thrombocytopenic and thromboembolic cases, after 22 million first doses and 6.8 million second doses of the ChAdOx1 vaccine^17^. Three separate case series have described patients who developed thrombocytopenic and thrombotic events after ChAdOx1 vaccination, which clinically mimics heparin-induced thrombocytopenia^11–13^. ChAdOx1’s summary of product characteristics has now been updated accordingly. The JCVI has recommended that 18–39-year-old individuals who do not have an underlying health condition should be offered an alternative to ChAdOx1, if available^18,19^. There have also been reports of post-vaccination exacerbation of chronic idiopathic or immune thrombocytopenic purpura among individuals receiving mRNA vaccines (including BNT162b2)^20–22^.

Given ongoing public, professional and regulatory concerns and the limited body of population-based evidence, there is an urgent need for evidence on the safety of all COVID-19 vaccines and, in particular, any association between COVID-19 vaccines and ITP and venous thromboembolic (including CVST) and arterial thromboembolic and hemorrhagic events. To investigate this, we used a national prospective COVID-19 surveillance cohort in Scotland, which consisted of linked databases containing individual patient-level data relating to vaccination status, virological reverse transcription polymerase chain reaction (RT–PCR) COVID-19, laboratory tests and clinical and mortality records covering 5.4 million people (~99% of the Scottish population).

## Results

**Extended Data Fig. 1:**
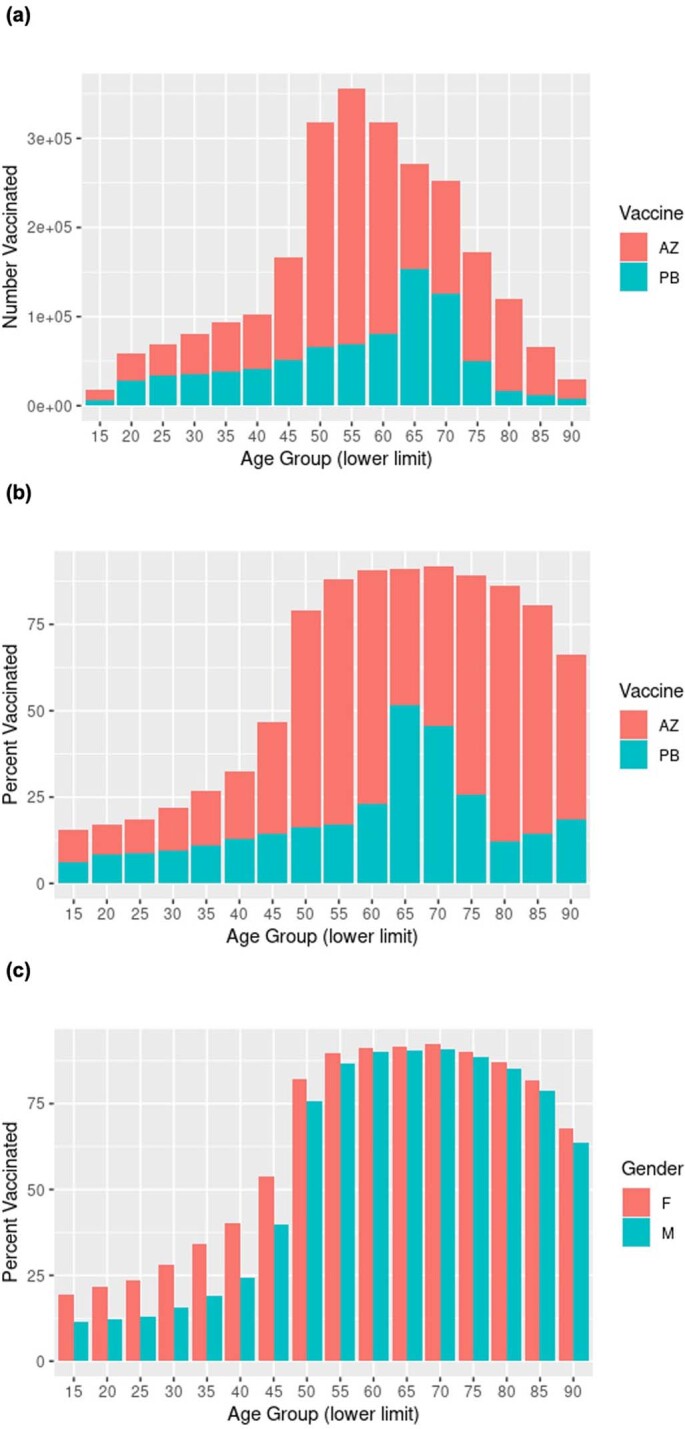
Vaccine uptake by type of vaccine for individuals in Scotland, up to 10th April 2020 - (a) number by vaccine type, (b) percentage of population by vaccine type, and (c) percentage uptake by age and sex. Note: (a) and (b) ChAdOx1 (AZ) or BNT162b2 (PB) vaccines, (c) both vaccines. The percentages are based upon the population alive at 8 December 2020. This is derived from the cohort set up on 1 March 2020 by removing individuals who died. Individuals in the population who died before vaccination will count in the denominator but cannot count in the numerator and this explains the drop off in uptake percentage in the elderly.

Between 8 December 2020 and 14 April 2021, 2.53 million people (57.5% of the adult population aged ≥18 years) received first doses of COVID-19 vaccines in Scotland. Of these, 1.71 milion people were vaccinated with the ChAdOx1 vaccine, and 0.82 million people were vaccinated with the BNT162b2 vaccine (Extended Data Fig. 1), with fewer than 10,000 people receiving the mRNA-1273 vaccine.

### Thrombocytopenic events

For thrombocytopenia events (excluding ITP), the aRR in the period 0–27 d after vaccination for ChAdOx1 vaccination was 1.42 (95% CI, 0.86–2.37). There was evidence of an increased risk of events 0–6 d after vaccination (aRR = 2.80, 95% CI, 1.39–5.67) (Table 1). In the adult population, the difference in expected versus observed events for thrombocytopenia was 1.33 (95% CI, −0.34–2.97) per 100,000 doses. The SCCS analysis, used as a post hoc analysis to investigate confounding by indication, for ChAdOx1 and thrombocytopenia (excluding ITP) found an RR of 1.07 (95% CI, 0.80–1.42) (Supplementary Table 3).

**Table 1:** Reported thrombocytopenic, venous and arterial thromboembolic and hemorrhagic events for the ChAdOx1 vaccine

Time period after vaccination	Number of individuals^a^	Number of events^b^	Unadjusted RR (95% CI)	Number of risk groups adjusted RR (95% CI)	Fully adjusted RR (95% CI)^c^
**Thrombocytopenia (excluding ITP)**					
Unvaccinated	2,343	207 (8.8%)	1.00 (1.00–1.00)	1.00 (1.00–1.00)	1.00 (1.00–1.00)
0–6 d	101	18 (17.8%)	3.54 (1.76–7.10)	2.76 (1.36–5.60)	2.80 (1.39–5.67)
7–13 d	127	8 (6.3%)	0.71 (0.28–1.77)	0.66 (0.27–1.60)	0.69 (0.29–1.67)
14–20 d	95	10 (10.5%)	1.55 (0.66–3.66)	1.12 (0.47–2.65)	1.20 (0.49–2.90)
21–27 d	91	10 (11.0%)	1.86 (0.75–4.58)	1.34 (0.55–3.28)	1.26 (0.51–3.14)
28+ d	407	48 (11.8%)	2.15 (1.18–3.94)	1.55 (0.84–2.88)	1.53 (0.82–2.87)
0–27 d	414	46 (11.1%)	1.82 (1.09–3.03)	1.40 (0.84–2.32)	1.42 (0.86–2.37)
**ITP**					
Unvaccinated	702	58 (8.3%)	1.00 (1.00–1.00)	1.00 (1.00–1.00)	1.00 (1.00–1.00)
0–6 d	35	≤5 (≤14.3%)	3.95 (1.08–14.52)	3.16 (0.82–12.19)	3.43 (0.88–13.33)
7–13 d	51	7 (13.7%)	4.51 (1.42–14.31)	4.31 (1.32–14.05)	4.60 (1.37–15.42)
14–20 d	39	7 (17.9%)	8.50 (2.53–28.57)	8.62 (2.55–29.07)	7.81 (2.28–26.71)
21–27 d	17	≤5(≤29.4%)	14.75 (2.67–81.51)	13.85 (2.44–78.62)	14.07 (2.46–80.31)
28+ d	48	≤5 (≤10.4%)	1.50 (0.27–8.19)	1.29 (0.22–7.54)	1.25 (0.21–7.46)
0–27 d	142	23 (16.2%)	6.01 (2.56–14.07)	5.67 (2.39–13.46)	5.77 (2.41–13.83)
**Venous thromboembolic events (including CVST)**					
Unvaccinated	26,843	2,449 (9.1%)	1.00 (1.00–1.00)	1.00 (1.00–1.00)	1.00 (1.00–1.00)
0–6 d	952	92 (9.7%)	1.13 (0.87–1.46)	0.97 (0.75–1.25)	0.96 (0.74–1.24)
7–13 d	1,074	101 (9.4%)	1.15 (0.89–1.49)	0.95 (0.73–1.22)	0.91 (0.71–1.18)
14–20 d	1,131	130 (11.5%)	1.61 (1.26–2.05)	1.30 (1.02–1.65)	1.23 (0.96–1.57)
21–27 d	953	100 (10.5%)	1.52 (1.16–2.00)	1.17 (0.89–1.54)	1.10 (0.84–1.44)
28+ d	3,616	381 (10.5%)	1.58 (1.30–1.93)	1.20 (0.98–1.47)	1.08 (0.88–1.32)
0–27 d	4,110	423 (10.3%)	1.32 (1.13–1.54)	1.08 (0.92–1.26)	1.03 (0.89–1.21)
**Arterial thromboembolic events**					
Unvaccinated	67,599	5,937 (8.8%)	1.00 (1.00–1.00)	1.00 (1.00–1.00)	1.00 (1.00–1.00)
0–6 d	3,211	303 (9.4%)	1.26 (1.09–1.46)	1.08 (0.94–1.26)	1.08 (0.93–1.25)
7–13 d	3,333	344 (10.3%)	1.54 (1.34–1.78)	1.26 (1.09–1.46)	1.25 (1.08–1.44)
14–20 d	3,352	350 (10.4%)	1.68 (1.45–1.95)	1.29 (1.11–1.50)	1.26 (1.09–1.46)
21–27 d	3,261	351 (10.8%)	1.89 (1.62–2.19)	1.40 (1.20–1.62)	1.37 (1.18–1.60)
28+ d	13,925	1,477 (10.6%)	2.03 (1.81–2.27)	1.37 (1.23–1.54)	1.33 (1.19–1.50)
0–27 d	13,157	1,348 (10.2%)	1.55 (1.41–1.70)	1.24 (1.13–1.36)	1.22 (1.12–1.34)
**Hemorrhagic events**					
Unvaccinated	8,972	785 (8.7%)	1.00 (1.00–1.00)	1.00 (1.00–1.00)	1.00 (1.00–1.00)
0–6 d	337	32 (9.5%)	1.39 (0.87–2.20)	1.22 (0.78–1.93)	1.08 (0.68–1.73)
7–13 d	350	44 (12.6%)	2.22 (1.47–3.37)	1.96 (1.29–2.98)	1.87 (1.23–2.84)
14–20 d	324	38 (11.7%)	2.09 (1.33–3.27)	1.80 (1.15–2.82)	1.67 (1.07–2.62)
21–27 d	314	35 (11.1%)	1.84 (1.16–2.89)	1.53 (0.97–2.42)	1.42 (0.90–2.25)
28+ d	1,316	129 (9.8%)	1.51 (1.06–2.14)	1.18 (0.82–1.68)	1.10 (0.77–1.57)
0–27 d	1,325	149 (11.2%)	1.86 (1.41–2.45)	1.60 (1.21–2.12)	1.48 (1.12–1.96)

^a^Number of individuals in the vaccine exposure group. ^b^Number of events is the number of individuals with an incident consultation in the post-vaccination period. Percent is the number of events divided by *n* and should be equal to 8.34% if there is no association between vaccination and the event representing the 10:1 ratio of controls to cases. ^c^Adjusted for number of clinical risk group, socioeconomic status and number of RT–PCR tests an individual had before 8 December 2020. *n* ≤ 5 denotes minimum allowable reported value.

No increased risk of thrombocytopenia events was found for the BNT162b2 vaccine (Table 2) during any of the post-vaccination time periods. There was no association found for the BNT162b2 vaccine in the SCCS analysis (Supplementary Table 3).

**Table 2:** Reported thrombocytopenic, venous and arterial thromboembolic and hemorrhagic events for the BNT162b2 vaccine

Time period after vaccination	Number of individuals^a^	Number of events^b^	Unadjusted RR (95% CI)	Number of risk groups adjusted RR (95% CI)	Fully adjusted RR (95% CI)^c^
**Thrombocytopenia (excluding ITP)**					
Unvaccinated	2,343	207 (8.8%)	1.00 (1.00–1.00)	1.00 (1.00–1.00)	1.00 (1.00–1.00)
0–6 d	43	≤5 (≤11.6%)	1.05 (0.29–3.78)	0.89 (0.25–3.23)	0.84 (0.23–3.08)
7–13 d	51	7 (14.6%)	2.10 (0.71–6.18)	1.71 (0.56–5.27)	1.80 (0.59–5.50)
14–20 d	34	0 (0.0%)	0.00 (0.00–Inf)	0.00 (0.00–Inf)	0.00 (0.00–Inf)
21–27 d	45	≤5 (≤11.1%)	0.32 (0.05–1.94)	0.35 (0.05–2.30)	0.35 (0.05–2.28)
28+ d	250	21 (8.4%)	1.06 (0.56–2.03)	0.91 (0.47–1.75)	0.80 (0.41–1.54)
0–27 d	173	13 (7.5%)	0.79 (0.37–1.67)	0.68 (0.32–1.45)	0.67 (0.32–1.43)
**ITP**					
Unvaccinated	702	58 (8.3%)	1.00 (1.00–1.00)	1.00 (1.00–1.00)	1.00 (1.00–1.00)
0–6 d	≤5	0 (0%)	0.00 (0.00–Inf)	0.00 (0.00–Inf)	0.00 (0.00–Inf)
7–13 d	15	≤5 (≤33.3%)	0.53 (0.04–8.05)	0.54 (0.03–8.41)	0.61 (0.04–9.28)
14–20 d	18	0 (0%)	0.00 (0.00–Inf)	0.00 (0.00–Inf)	0.00 (0.00–Inf)
21–27 d	11	≤5 (≤45.5%)	1.31 (0.16–10.45)	1.41 (0.18–11.31)	1.46 (0.18–12.01)
28+ d	49	5 (10.2%)	1.52 (0.47–4.88)	1.49 (0.45–4.91)	1.68 (0.48–5.87)
0–27 d	45	≤5 (≤5.8%)	0.44 (0.08–2.36)	0.50 (0.09–2.76)	0.54 (0.10–3.02)
**Venous thromboembolic events (including CVST)**					
Unvaccinated	26,843	2,449 (9.1%)	1.00 (1.00–1.00)	1.00 (1.00–1.00)	1.00 (1.00–1.00)
0–6 d	460	26 (5.7%)	0.46 (0.29–0.72)	0.43 (0.27–0.67)	0.40 (0.26–0.63)
7–13 d	589	38 (6.5%)	0.54 (0.36–0.81)	0.49 (0.33–0.74)	0.45 (0.30–0.67)
14–20 d	547	37 (6.8%)	0.59 (0.39–0.89)	0.52 (0.34–0.79)	0.48 (0.32–0.73)
21–27 d	448	36 (8.0%)	0.80 (0.52–1.22)	0.72 (0.47–1.10)	0.64 (0.42–0.97)
28+ d	2,299	203 (8.8%)	1.02 (0.83–1.25)	0.88 (0.72–1.08)	0.73 (0.60–0.90)
0–27 d	2,044	137 (6.7%)	0.58 (0.47–0.73)	0.53 (0.42–0.66)	0.48 (0.39–0.61)
**Arterial thromboembolic events**					
Unvaccinated	26,843	2,449 (9.1%)	1.00 (1.00–1.00)	1.00 (1.00–1.00)	1.00 (1.00–1.00)
0–6 d	460	26 (5.7%)	0.46 (0.29–0.72)	0.43 (0.27–0.67)	0.40 (0.26–0.63)
7–13 d	589	38 (6,5%)	0.54 (0.36–0.81)	0.49 (0.33–0.74)	0.45 (0.30–0.67)
14–20 d	547	37 (6.8%)	0.59 (0.39–0.89)	0.52 (0.34–0.79)	0.48 (0.32–0.73)
21–27 d	448	36 (8.0%)	0.80 (0.52–1.22)	0.72 (0.47–1.10)	0.64 (0.42–0.97)
28+ d	2,299	203 (8.8%)	1.02 (0.83–1.25)	0.88 (0.72–1.08)	0.73 (0.60–0.90)
0–27 d	2,044	137 (6.7%)	0.58 (0.47–0.73)	0.53 (0.42–0.66)	0.48 (0.39–0.61)
**Hemorrhagic events**					
Unvaccinated	8,972	785 (8.7%)	1.00 (1.00–1.00)	1.00 (1.00–1.00)	1.00 (1.00–1.00)
0–6 d	132	16 (12.1%)	1.69 (0.92–3.09)	1.40 (0.76–2.58)	1.10 (0.59–2.06)
7–13 d	157	13 (8.3%)	1.08 (0.56–2.09)	0.93 (0.47–1.81)	0.76 (0.39–1.50)
14–20 d	189	21 (11.1%)	1.62 (0.91–2.88)	1.40 (0.79–2.50)	1.27 (0.71–2.27)
21–27 d	169	11 (6.5%)	0.79 (0.39–1.57)	0.70 (0.35–1.41)	0.60 (0.30–1.22)
28+ d	762	76 (10%)	1.44 (1.04–2.00)	1.26 (0.90–1.76)	1.07 (0.76–1.51)
0–27 d	647	61 (9.4%)	1.25 (0.89–1.77)	1.08 (0.77–1.53)	0.92 (0.64–1.30)

Inf, infinity.

^a^Number of individuals in the vaccine exposure group. ^b^Number of events is the number of individuals with an incident consultation in the post-vaccination period. Percent is the number of events divided by *n* and should be equal to 9.1% if there is no association between vaccination and the event representing the 10:1 ratio of controls to cases. ^c^Adjusted for number of clinical risk group, socioeconomic status and number of RT–PCR tests an individual had before 8 December 2020. *n* ≤ 5 denotes minimum allowable reported value.

### ITP

For ITP, the aRR in the period 0–27 d after vaccination for ChAdOx1 was 5.77 (95% CI, 2.41–13.83). This represents an estimated incidence of 1.13 (0.62–1.63) cases per 100,000 doses. This increased risk was first found at 7–13 d after vaccination (aRR = 4.60, 95% CI, 1.37–15.42) and was most pronounced at 21–27 d (aRR = 14.07, 95% CI, 2.46–80.31) (Table 1). The wide CIs reflect the small number of incident ITP cases over the study period. The SCCS post hoc analysis RR for ChAdOx1 vaccination and ITP (28 d after vaccination versus 14 d and 104 d before vaccination) was 1.98 (95% CI, 1.29–3.02) (Supplementary Tables 3 and 4). The difference in expected versus observed events for ITP during the post-ChAdOx1 vaccination period for 40–49-year-old individuals was 0.62 events (95% CI, 0.01–1.36) per 100,000 doses (Table 3). In the adult population studied, the difference in expected versus observed events was 0.46 events (95% CI, −0.44–1.33) per 100,000 doses.

**Table 3:** Observed versus expected thrombocytopenia and ITP events after COVID-19 vaccination

Age group	Number study individuals vaccinated	Thrombocytopenia (excluding ITP)			ITP		
		Observed events^a^	Expected number of events^b^	Observed–expected (95% CI)^c^	Observed events^a^	Expected number of events^b^	Observed–expected (95% CI)^c^
**ChAdOx1**							
16–39	181,635	≤5^d^	≤5^d^	1.17 (−1.83–5.13)	≤5^d^	≤5^d^	2.62 (−0.38–6.62)
40–59	740,800	22	14.3	7.71 (−2.71–18.63)	6	1.4	4.58 (0.10–10.05)
60–79	606,854	49	40.6	8.41 (−10.08–27.10)	14	7.1	6.85 (−2.23–15.96)
80+	178,673	32	26.6	5.40 (−12.89–23.00)	5	11.3	−6.26 (−16.76–3.25)
**BNT162b2**							
16–39	144,393	7	3.8	3.23 (−1.85–9.30)	≤5^d^	≤5^d^	1.20 (−1.13–4.47)
40–59	230,802	6	13.0	−7.00 (−13.59–0.07)	≤5^d^	≤5^d^	(−0.33(−2.66–2.23)
60–79	409,429	27	34.2	−7.24 (−21.63–7.54)	≤5^d^	≤5^d^	−2.05 (−8.24–3.81)
80+	36,428	≤5^d^	≤5^d^	−3.38 (−8.02–1.53)	0	2.7	−2.70 (−4.96–0.90)

^a^The observed events were incident cases after vaccination counted for the observed duration of the post-vaccination period. ^b^The expected events are the number of events per day in the pre-vaccination period divided by the population and multiplied by the days at risk in the post-vaccination period for all vaccinated and then summed. ^c^The difference between expected (the number of events per day in the pre-vaccination period divided by the population and multiplied by the days at risk in the post-vaccination period for all vaccinated and then summed) and observed events during the post-vaccination period, with CIs obtained from a parametric bootstrap, was based on the Poisson distribution using 10,000 samples. There was limited opportunity for matching in this analysis, and the findings, therefore, need to be interpreted with caution. ^d^*n* ≤ 5 denotes minimum allowable reported value.

Patients who had ITP after ChAdOx1 vaccination compared to those who were unvaccinated at the time of the event tended to be older (median age, 69 years versus 54 years, *P* = 0.01), to be more likely to have at least one clinical risk condition (85% compared to 40%, *P* = 0.001) and to have been in hospital at the time of the event (52% versus 28%, *P* = 0.05). The gender distribution was the same for ITP after ChAdOx1 vaccination compared to those who were unvaccinated. For the 22 patients with post-vaccination ITP and for whom platelet counts were available after vaccination, all but two had counts below 100,000 per µl. In addition, 48% of patients with post-ChAdOx1 ITP had prior prescriptions that could induce ITP, compared to 35% of those who were unvaccinated at the time of their ITP event. Five or fewer (≤10%) patients with ITP were prescribed ITP therapies by general practitioners in the community after vaccination with ChAdOx1.

No positive association was found between the BNT162b2 vaccine and ITP (Table 2): aRR at 0–27 d after vaccination was 0.54 (95% CI, 0.10–3.02). There was also no clear evidence of association found for the BNT162b2 vaccine in the SCCS analysis (Supplementary Table 3).

In total, three deaths were reported after ITP. These deaths occurred in both vaccinated and unvaccinated individuals all aged over 70 and for reasons not associated with ITP.

**Extended Data Fig. 2:**
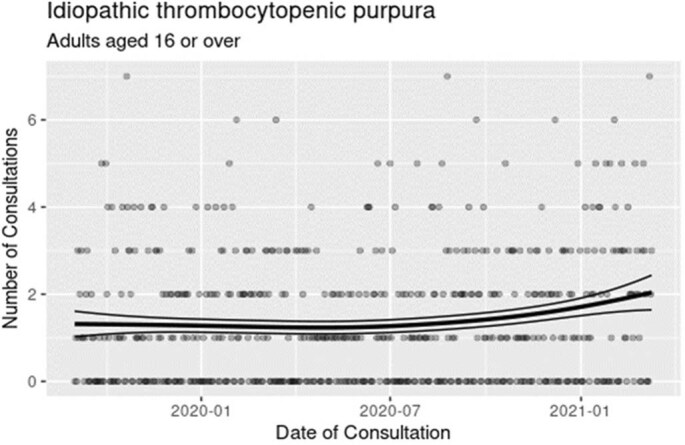
Number of ITP events per day since September 2019. Note: The line comes from a generalised additive Poisson model fitted to the number of cases with a simple spline term for the trend. Graph based upon data up to 22 March 2020. One of the assumptions of the standard self-controlled case series analysis is that the underlying rates of the event are constant over time. Fig. S2 shows the number of events for idiopathic thrombocytopenic purpura (ITP) since September 2019. A generalised Additive Poisson model was fitted to the daily data with a spline for the number of days since 1 September 2019 and a factor for the months. There was no evidence of any seasonal pattern associated with the months of the year (p = 0.189, using a change in deviance test) but there was evidence of a non-linear trend (p = 0.0138). Further investigation of the trend was undertaken by including binary change in level variables associated with the period post BNT162b2 (8 December 2020) and post ChAdOx1 (5 January 2021) This showed that there was no residual increasing trend (p = 0.58), nor any change in level following the introduction of BNT162b2 (p = 0.4315) but there was evidence of a change in level amounting to an 0.44 (95% CI 0.20, 0.68; p = 0.0003), additional events per day in the period after the introduction of the ChAdOx1 vaccine.

**Extended Data Fig. 3:**
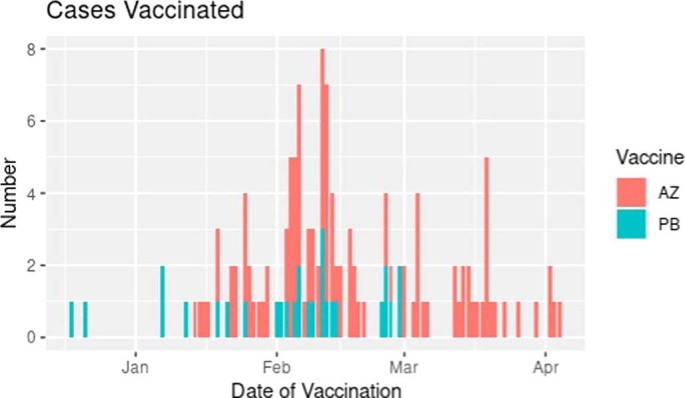
Dates of vaccination and type of vaccine for individuals with an ITP event during the study period. Note: Figure shows when individuals with an ITP event were vaccinated. Most received the ChAdOx1 (AZ) rather than the BNT162b2 (PB) vaccine.

The number of events per day for ITP since September 2019 is available in Extended Data Fig. 2, showing stable rates until January 2021 when the ChAdOx1 vaccine was introduced in the UK, followed by an increase in the number of events per day. Dates of vaccination and type of vaccine for individuals with an ITP event during the study period are available in Extended Data Fig. 3.

### Venous thromboembolic events

We found no association between prior ChAdOx1 vaccination and venous thromboembolic events (including CVST) at 0–27 d after vaccination (aRR = 1.03, 95% CI, 0.89–1.21) or for the BNT162b2 vaccination 0–27 d after vaccination (aRR = 0.50, 95% CI, 0.40–0.62). No increase in the odds of venous thromboembolic events was found during any of the post-vaccination time periods analyzed for ChAdOx1 (Table 1) or BNT162b2 (Table 2) vaccines. The SCCS analysis for ChAdOx1 and venous thromboembolic events found an RR = 0.94 (95% CI, 0.87–1.02) (Supplementary Table 3). No association was found in the SCCS analysis for the BNT162b2 vaccine (Supplementary Table 3)

The difference between observed versus expected cases for younger age groups after ChAdOx1 vaccination was 10.41 (95% CI, 4.64–16.65) per 100,000 doses for 16–39-year-old individuals and 9.60 (95% CI, 5.57–13.69) per 100,000 doses for 40–59-year-old individuals (Table 4). In the adult population included in the study, the difference in expected versus observed events was 4.46 events (95% CI, −0.59–9.43) per 100,000 doses.

**Table 4:** Observed versus expected venous and arterial thromboembolic and hemorrhagic events after COVID-19 vaccination

Age group	Number study individuals vaccinated	Venous thromboembolic events (including CVST)			Arterial thromboembolic events			Hemorrhagic events		
		Observed events^a^	Expected number of events^b^	Observed–expected (95% CI)	Observed events^a^	Expected number of events^b^	Observed–expected (95% CI)	Observed events^a^	Expected number of events^b^	Observed–expected (95% CI)
**ChAdOx1**										
16–39	181,635	30	11.1	18.90 (8.42–30.25)	15	4.3	10.67 (3.33–18.81)	9	5.0	4.04 (−1.57–10.47)
40–59	740,800	178	106.9	71.11 (41.66–100.51)	516	283.8	232.23 (182.63–282.63)	58	41.9	16.13 (−0.85–34.07)
60–79	606,854	439	423.4	15.60 (−44.53–74.57)	1,600	1,521.0	78.96 (−32.80–188.70)	131	149.9	−18.85 (−52.50–14.35)
80+	178,673	246	275.3	−29.28 (−83.56–25.40)	1,157	1,519.0	−362.03 (−488.57–239.94)	103	151.9	−48.95 (−87.64–10.46)
**BNT162b2**										
16–39	144,393	24	22.9	1.09 (−9.34–12.21)	5	8.9	−3.90 (−8.95–1.79)	10	10.2	−0.24 (−6.86–7.11)
40–59	230,802	80	97.3	−17.29 (−38.85–4.78)	217	258.0	−41.00 (−76.37–4.39)	37	38.1	−1.11 (−15.32–13.30)
60–79	409,429	259	357.2	−98.22 (−144.82–51.85)	1,156	1,283.4	−127.38 (−222.72–35.17)	88	126.4	−38.41 (−65.85–11.24)
80+	36,428	58	66.0	−8.00 (−26.38–11.01)	225	364.0	−138.96 (−177.19–99.42)	29	36.4	−7.43 (−20.35–6.31)

^a^The observed events were incident cases after vaccination counted for the observed duration of the post-vaccination period. ^b^The expected events are the number of events per day in the pre-vaccination period divided by the population and multiplied by the days at risk in the post-vaccination period for all vaccinated and then summed. ^c^The difference between expected (the number of events per day in the pre-vaccination period divided by the population and multiplied by the days at risk in the post-vaccination period for all vaccinated and then summed) and observed events during the post-vaccination period, with CIs obtained from a parametric bootstrap, was based on the Poisson distribution using 10,000 samples. There was limited opportunity for matching in this analysis, and the findings, therefore, need to be interpreted with caution.

A subgroup analysis of deep vein thrombosis (DVT) and pulmonary embolism (PE) found no clear association with ChAdOx1 vaccination (0–27 d after vaccination: DVT: aRR = 1.21, 95% CI, 0.95–1.54; PE: aRR = 0.78, 95% CI, 0.63–0.96) or BNT162b2 vaccination (0–27 d after vaccination: DVT: aRR = 0.79, 95% CI, 0.56–1.11; PE: aRR = 0.35, 95% CI, 0.26–0.48).

### CVST

When focusing on CVST events, 19 total incident events were recorded among all people (vaccinated and unvaccinated). Among those vaccinated before the incident event, there were insufficient events to adequately power an analysis (*n* = 6). Post-vaccination incident events were recorded for both vaccines. Two of those individuals with a post-vaccination CVST event died. Platelet count results were available for 17 of 19 individuals identified as having CVST. There was no evidence of platelet counts <150,000 per µl at any time point in any of the 17 individuals with a post-vaccination CVST event.

### Arterial thromboembolic events

At 0–27 d after vaccination, increased risk between ChAdOx1 vaccination and arterial thromboembolic events was found (aRR = 1.22, 95% CI, 1.12–1.34) (Table 1). This was first seen at 7–13 d after vaccination (aRR = 1.25, 95% CI, 1.08–1.44) and also at 14–20 d after vaccination (aRR = 1.26, 95% CI, 1.09–1.46). For all ages combined, fewer events were observed during the post-vaccination period (3,288) compared to 3,328 expected (Table 4). The SCCS analysis RR for ChAdOx1 and arterial thromboembolic events was 0.97 (95% CI, 0.93–1.02) (Supplementary Table 3).

The observed versus expected cases for younger age groups were 5.87 (95% CI, 1.83–10.36) per 100,000 doses for 16–39-year-old individuals and 31.35 (95% CI, 24.69–38.08) per 100,000 doses for 40–59-year-old individuals (Table 4).

There was no increased risk of arterial thromboembolic events associated with BNT162b2 vaccination at 0–27 d after vaccination (aRR = 0.92, 95% CI, 0.81–1.04) or during any of the post-vaccination time periods analyzed (Table 2 and Supplementary Table 3).

### Hemorrhagic events

At 0–27 d after vaccination, increased risk between ChAdOx1 vaccination and hemorrhagic events was found (aRR = 1.48, 95% CI, 1.12–1.96) (Table 1). This was first seen at 7–13 d after vaccination (aRR = 1.87, 95% CI, 1.23–2.84) and also at 14–20 d after vaccination (aRR = 1.67, 95% CI, 1.07–2.62). For all ages combined, fewer events were observed during the post-vaccination period (301 events) compared to 349 expected events (Table 4). The SCCS analysis (used to investigate confounding by indication) found no clear evidence of associations between ChAdOx1 (RR = 0.95, 95% CI, 0.82–1.11) and hemorrhagic events (Supplementary Table 3).

The observed versus expected cases for younger age groups were 2.22 (95% CI, −0.82–5.81) per 100,000 doses for 16–39-year-old individuals and 2.18 (95% CI, -0.11–4.60) per 100,000 doses for 40–59-year-old individuals (Table 4).

There was no increased risk of hemorrhagic events associated with BNT162b2 vaccination at 0–27 d after vaccination (aRR = 0.92, 95% CI, 0.64–1.30) or during any of the post-vaccination time periods analyzed (Table 2). There was also no clear evidence of association found for the BNT162b2 vaccine (Supplementary Table 3).

### Multiple outcomes and predictors

Overlap in our outcomes with individuals presenting with multiple events of interest was rare. Among those vaccinated with ChAdOx1, there were six occurrences of ITP with hemorrhagic, venous thromboembolic or thrombocytopenia events, with the most common combination being ITP and thrombocytopenia.

We found increased risk for post-vaccination ITP and arterial thromboembolic and hemorrhagic events combined associated with increasing age (especially over 60), male sex, having certain comorbidities (such as heart failure, coronary heart disease, peripheral vascular disease, severe mental illness, sickle cell disease, prior stroke, type 1 and 2 diabetes and chronic kidney disease (stage 5)), very high blood pressure and smoking (Supplementary Table 5).

### Sensitivity analyses

Restricting the end date of analysis to 21 February 2021 for ITP and hemorrhagic events resulted in fewer ChAdOx1-associated events and similar RRs with wider CIs (ITP: aRR = 8.80, 95% CI, 0.70–108.00; arterial thromboembolic event: aRR = 1.11, 95% CI, 0.96–1.28; hemorrhagic event: aRR = 1.16, 95% CI, 0.71–1.90). Restricting to those who never tested positive previously for severe acute respiratory syndrome coronavirus 2 (SARS-CoV-2) revealed similar RRs (ITP: 6.06, 95% CI, 2.28–16.12; arterial thromboembolic: 1.28, 95% CI, 1.16–1.41; hemorrhagic event: 1.57; 95% CI, 1.17–2.10). Additional adjustment for specific health conditions did not substantively change the estimated associations for ChAdOx1 and ITP (Supplementary Table 2).

## Discussion

This Scottish national population-based analysis among 2.53 million people who received their first doses of SARS-CoV-2 vaccines reveals a potential association between receiving a first-dose ChAdOx1 vaccination and occurrence of ITP, with an incidence of 1.13 cases per 100,000 vaccinations. For ChAdOx1 vaccination, there was suggestive evidence of an association with arterial thromboembolic and hemorrhagic events. For these outcomes, an attenuation of effect was found in the SCCS analysis, which might indicate the presence of residual confounding or confounding by indication. For any venous thromboembolic event, there were more observed than expected events for younger age groups (16–59 years old) associated with ChAdOx1, but this was not seen in our primary incident case–control analysis. Because of our limited ability to match in the observed versus expected analysis, this finding should be treated with considerable caution. There were 19 incident CVST events seen in our study population: 6 of these occurred after vaccination, with these events being seen after both ChAdOx1 and BNT162b2 vaccines. Two individuals with post-vaccination CVST events died. For CVST (and other rare conditions), there were insufficient numbers to draw any reliable conclusions other than, if there is any association, it is likely to represent an extremely rare outcome. For the BNT162b2 vaccine, our analysis found no evidence of increased adverse events for the thrombocytopenic, venous thromboembolic or hemorrhagic outcomes of interest.

To our knowledge, this is the one of the first real-world contemporaneous studies identifying all vaccinated individuals within a national population and assessing COVID-19 vaccine-related thrombocytopenic, venous or arterial thromboembolic and hemorrhagic adverse events. One published study of people aged 18–65 years who received the ChAdOx1 vaccine in Denmark and Norway observed increased rates of venous thromboembolic events, including cerebral venous thrombosis (standardized morbidity ratio of 1.97 and 95% CI, 1.50–2.54) and intracerebral hemorrhage (standardized morbidity ratio of 2.33 and 95% CI, 1.01–4.59)^23^. In that study, Pottegård et al. found a standardized morbidity ratio for any thrombocytopenia/coagulation disorders of 1.52 (0.97–2.25) and for any bleeding of 1.23 (0.97–1.55).

Our study has several strengths, including our ability to rapidly access and analyze data on vaccination status and medical and death records from linked national databases^24–26^. This study is, therefore, less susceptible to recall or misclassification bias than studies of samples of the population. A large population aided study power to facilitate the analysis of rare events such as ITP. We think that our findings have generalizability across countries using these vaccines as part of national vaccination programs that have prioritized vaccination of high-risk populations.

Our study has several limitations. As few individuals had received two vaccine doses at the time of analysis, this (second-dose) subgroup was not investigated separately. A further analysis on second doses will be conducted in due course. Furthermore, our study included few young vaccinated people (<40 years), especially for the ChAdOx1 vaccine, because the vaccination program has been predominantly targeted by age and underlying comorbidities so far. Although electronic general practice records of hospitalization and deaths were accessible, and linked with hospitalization and mortality records, lags in final coded hospital discharge data and postmortem changes to death certification might have resulted in over-riding of initial recorded causes of hospitalizations and deaths in some instances. However, our sensitivity analysis restricting to an earlier date of follow-up is less subject to such potential biases and found similar results. Additionally, ITP is a diagnosis of exclusion. Given that we based this analysis on clinician-recorded data, we had to assume that clinicians had appropriately investigated patients for their thrombocytopenia before recording this diagnosis. Discussions with Scottish hematologists indicated that this was a reasonable assumption, as the diagnosis of ITP is made only by specialists in a Scottish context. However, there can be uncertainty about the diagnosis of ITP, and published experience indicates that the diagnosis of ITP is often changed when patients are followed by skilled hematologists^27,28^. There is also the possibility that some of these cases of ITP could have represented reactivation of disease that had been in remission for more than 1 year. We did, however, carry out a post hoc analysis of all post-vaccination ITP events for those with prior available platelet count (tested in the primary care setting) and relevant prescriptions that could cause thrombocytopenia. We also carried out an analysis of ITP-directed therapy after vaccination (including oral corticosteroids). We were unable to access blood smear information as this is not routinely captured in the record systems that we had access to. Furthermore, 48% of patients with post-ChAdOx1 ITP events had prior prescriptions that could induce ITP, compared to 35% of those who were unvaccinated at the time of their ITP event. ITP-directed therapy prescribed by general practitioners in the community to patients with post-vaccination ITP was uncommon (≤10%). The overwhelming majority of ITP-directed therapy (for example, pulsed dexamathasone, prednisolone with or without intravenous immunoglobulin, rituximab and immunosuppressants) is, however, is likely to have been initiated in the hospital setting by hematologists, but these data were not accessible to us, as hospital prescribing in Scotland remains predominantly paper based^29^. These patients with community prescribing of oral corticosteroids are likely to have had persistent ITP that was managed in primary care.

Although we used a nested case–control study design matched by age, sex and geography, and adjusted for several confounders, unmeasured confounders could still have influenced our estimates (Supplementary Table 2). To mitigate this risk, where associations between ChAdOx1 and any adverse event were seen (that is, ITP and arterial thromboembolic and hemorrhagic events), we conducted a confirmatory post hoc SCCS analysis. SCCS designs can account for time-invariant confounding but are less suitable where recurrent events are not independent. Although the pattern of findings was largely similar across different analytical approaches, it is worth noting that the magnitude of associations did differ. Estimates tended to be greater in the case–control analysis, which could arise from potential residual confounding by indication that would most likely result in an overestimate of the real effect sizes. By contrast, the SCCS analysis tended to estimate smaller effect sizes, but the potential correlation of outcomes within an individual over time could bias estimates toward the null. The two approaches, therefore, provide reasonable bounds for the true effect, with our primary results potentially overestimating the risk of vaccine-associated harm and, therefore, being the most conservative for decision-making. Owing to the small number of adverse events, to identify predictors among vaccinated individuals we combined the outcomes of interest: ITP and arterial thromboembolic and hemorrhagic events. This as an area for future work, for instance through a meta-analyses of vaccine safety studies. Finally, the EAVE II platform is a national public health surveillance platform that was established at the request of the Scottish Government to help inform the public health response to the pandemic. It brought together a range of national whole-population healthcare datasets for the first time into Public Health Scotland. Ethical permission for this study was granted, and the Public Benefit and Privacy Panel Committee of Public Health Scotland approved the linkage and analysis of the de-identified datasets. As the policy aim was for national coverage, it was not feasible to obtain individual patient consent. This, therefore, restricted our ability to interrogate and report on certain individual record data in detail. For CVST, for instance, there were very few events, and, in keeping with our permissions, we suppressed the actual number of events found to minimize the risk of inadvertent disclosure of identity^30^. Also, centralized adjudication of our outcomes through case record review by an independent group of experts was not possible because access to data was limited to a small number of approved researchers.

The Centers for Disease Control and Prevention estimated that 60,000–100,000 Americans die annually due to venous thromboembolism (United States of America: 2.8 million deaths annually)^31^. Venous thromboembolic events are common in patients with COVID-19. Approximately 10% of patients with COVID-19 in hospitals (non-intensive care unit (ICU)) are diagnosed with venous thromboembolism and 28% of those in ICU^11,32,33,34^. The vaccine-induced adverse events after administration of the adenovirus-based SARS-CoV-2 vaccines (including the ChAdOx1 vaccine) have been described as vaccine-induced immune thrombotic thrombocytopenia (VITT) syndrome or thrombosis with thrombocytopenia syndrome resulting in a venous or arterial thrombosis, including CVST and thrombocytopenia^35^. The syndrome has been characterized as being similar to heparin-induced thrombocytopenia, a pro-thrombotic adverse drug reaction caused by the transient production of platelet-activating antibodies of IgG class that recognize multi-molecular complexes of (cationic) platelet factor 4 bound to (polyanionic) heparin^36^. We were insufficiently powered to provide estimates of the rarer VITT CVST and splanchnic vein thrombosis. This is an area for further work likely best pursued through larger datasets and meta-analyses.

ITP has also emerged as an important complication of COVID-19, with early epidemiological evidence suggesting a rate of 0.34% among hospitalized patients. There have also been reports of post-vaccination ITP in patients who received mRNA vaccines (including BNT162b2), and it has been postulated that some individuals might have had mild ‘compensated’ thrombocytopenia of diverse causes, and severe thrombocytopenia might have been induced by enhancement of macrophage‐mediated clearance or impaired platelet production as part of a systemic inflammatory response to vaccination^21^. ITP, however, as an adverse event after vaccine administration, is very rare. Our study suggests that there might be an increase in the risk of this very rare outcome for ChAdOx1 that is similar to other vaccines, including hepatitis B; measles, mumps and rubella; and influenza^37,38^. This very small risk is important but needs to be seen within the context of the very clear benefits of the ChAdOx1 vaccine.

As a result of findings from UK pharmacovigilance and surveillance data (including from EAVE II investigators), advice was issued in April 2021 regarding age group limits for the ChAdOx1 vaccine for individuals younger than 30 years of age^18^ and then, in May 2021, for individuals younger than 40 years of age^19^. Replication of our study in other countries is needed to confirm our results. We plan to update our analysis as the vaccine program is extended to younger, healthier individuals and as new vaccines become available. We also plan to extend our pharmacovigilance efforts to cover the second doses of these and other vaccines.

In conclusion, we did not identify any overall increased risk in the adverse events of interest in individuals receiving BNT162b2. First dose of ChAdOx1 was found to be associated with small increased risks of ITP, with suggestive evidence of an increased risk of arterial thromboembolic and hemorrhagic events. Given these small increased risks for ChAdOx1, alternative vaccines for individuals at low COVID-19 risk might be warranted when supply allows.

**Fig. 1:**
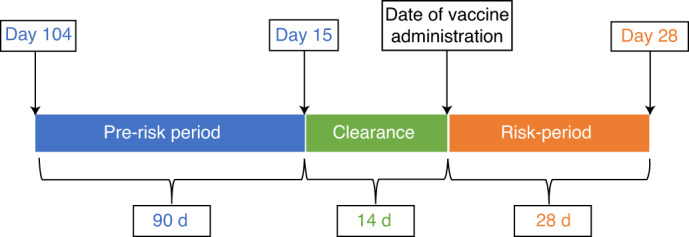
Schematic presentation of the self-controlled case series study design. Blue, the control (pre-risk) period: the 90-day period prior to 14 d before vaccination (i.e., 15–104 d before vaccine receipt). Green, a 14-day clearance period. Orange, the time period from the date of first vaccination dose to 28 d after, as the risk (exposed) period.

## Methods

### Ethics and permissions

Ethical permission for this study was granted by the South East Scotland Research Ethics Committee 02 (12/SS/0201). The Public Benefit and Privacy Panel Committee of Public Health Scotland approved the linkage and analysis of the de-identified datasets for this project (1920–0279).

### Study setting and population

The National Health Service in Scotland (NHS Scotland) provides comprehensive health services that are free at the point of care for all residents. Our base population for this study was 5.4 million residents (~99% of the population) registered with a general medical practice in Scotland.

### Study design

Following a pre-specified analysis plan, we started with a matched case–control study nested within the EAVE II prospective cohort. A case was defined as anyone with a recorded incident event of thrombocytopenia, venous thromboembolism, arterial thromboembolism or hemorrhage after the start of the COVID-19 vaccination program in Scotland. The general practice clinical data contained records of the specified events from 1 September 2019 to 14 April 2021. An incident case was defined as the first event in the period from when vaccination started on 8 December 2020 with no prior thrombocytopenic, venous thromboembolic or hemorrhagic clinical events since 1 September 2019. We matched cases who had experienced the outcomes of interest during the period from 8 December 2020 to 14 April 2021 based on recording of demographic and pre-existing comorbidities (Supplementary Table 2) to controls who had not yet experienced the outcome in an incident-matched nested case–control design. Incident cases were matched to controls based on age (in exact years up to 80 years old and then 2-year age groups up to 90 years old and 5-year age bands above that to allow for sparse data), sex and area of residence using Intermediate Zone classification. There are 1,271 such zones in Scotland (comprising between 2,500 and 6,000 households, with an average population of 4,200), which are geographically aligned with general practices. Ten controls were selected per case. Diagnosis dates of the cases were taken as the index dates for the controls.

We investigated confounding by indication and carried out a post hoc SCSS analysis to estimate the risk of COVID-19 vaccination and ITP events using conditional logistic regression with an offset for the length of the risk period^39^.

### Data sources

**Extended Data Fig. 4:**
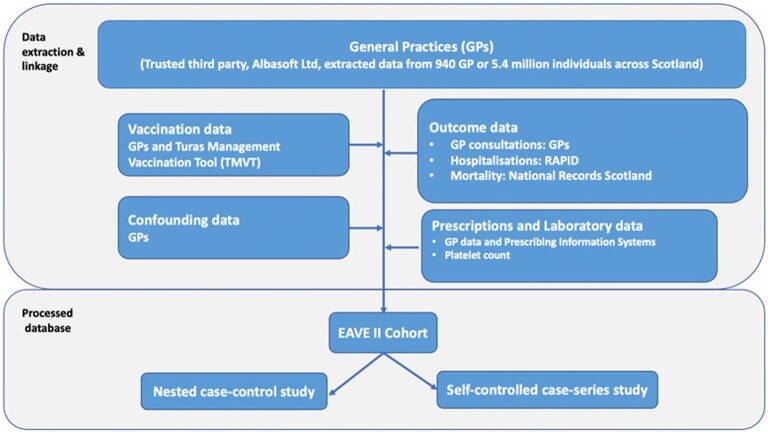
Data linkage diagram. Note: Community Health Index (CHI) numbers were used to link all datasets. Details on these datasets are available in our published protocol (Simpson CR, Robertson C, Vasileiou E, et al. Early Pandemic Evaluation and Enhanced Surveillance of COVID-19 (EAVE II): protocol for an observational study using linked Scottish national data. BMJ Open 2020;10:e039097. doi: 10.1136/bmjopen-2020-039097) There are two core methods of recording vaccine delivery, the national Turas Management Vaccination Tool (TMVT) system and local GP IT systems. TMVT was developed as a web application by National Education Scotland (NES). It is in general the preferred method of recording a vaccination where this is done outside the normal vaccine locations, predominantly dedicated vaccination centres and community programmes. Most vaccinations delivered in general practice settings are recorded in local IT systems; there are a few geographical areas that have however mandated the use of TMVT in every setting, including GP practices. Currently GP’s are paid per 100 vaccines administered so they are highly motivated to record information accurately. If this is not recorded to a minimum standard, they will not receive payment. All vaccines administered through vaccination centres and community programmes are accounted for on a daily basis. All vaccines recorded via TMVT are transferred to the national clinical datastore (NCDS) then to Albasoft on a daily basis. At 9 pm each night, these are loaded into a secure database and each practice “polls” the data store as part of the ESCRO data pump run between 12:00am and 5:00am each day to request the records for their specific practice. These are then loaded into a local queue at the practice for processing later in the day. As part of the same data pump run, the local GP IT system is queried and all vaccination records for the previous day are extracted (with a 10 day overlap to catch any retrospective recording) These records are then transferred back to Albasoft and collated into a single data source which is returned to the National Clinical Data Store (NCDS) at 8am each morning. As a result, all vaccinations recorded either by TMVT or GP IT systems pass through Albasoft in a 24-hour cycle. As part of the agreement to provide these data for EAVE II, vaccination records from both the TMVT and GP IT systems are transferred each day following the National Clinical Data Store processing to the EAVE II secure datastore in Public Health Scotland (PHS). This ensures that the EAVE II data are as up to date as possible. It is therefore extremely unlikely that any vaccinations will have been missed. Prescriptions: Glycoprotein IIb/IIIa inhibitors (tirofiban, abciximab, and eptifibatide), heparin, cephalosporins, linezolid, penicillins, sulphonamides, trimethoprim, vancomycin, quinine, ethambutol, rifampicin, carbamazepine, phenytoin, sodium valproate, ibuprofen, naproxen,, amiodarone, furosemide, quinidine, thiazides,, haloperidol, paracetamol, irinotecan, mirtazapine, oxaliplatin, salmeterol, tacrolimus and ciprofloxacin (prescription inducing thrombocytopenia); oral corticosteroids, intravenous immunoglobulin, azathioprine, ciclosporin, cyclophosphamide, danazol, dapsone, mycophenolate, rituximab, vinca alkaloids, eltrombopag, romiplostim (ITS related prescribing).

Almost all residents in Scotland are registered with a general practice and have a unique Community Health Index (CHI) number used by NHS Scotland. We used the CHI number to deterministically link all datasets with vaccination records in Public Health Scotland (Extended Data Fig. 4). Vaccination information was extracted from the general practice records and the Turas Vaccination Management Tool system; together, these captured all vaccination records, including those vaccinated in general practices, community vaccination hubs and other settings, such as care homes and hospitals in Scotland^40^. Further details on the data sources used in this study are available in a published project protocol^24^.

### Exposure definition

We studied the first doses of the BNT162b2 (ref. ^41^) and ChAdOx1 (ref. ^42^) vaccines. An individual was defined as exposed to each vaccine if they received their first dose of vaccine between 8 December 2020 and 14 April 2021. Given the limited number of people who had received their second vaccine dose by this time, we have reported only on first-dose-associated events.

### Outcomes

The outcomes analyzed in this study were one or more (1) thrombocytopenic, (2) arterial or venous thromboembolic or (3) hemorrhagic (excluding traumatic, gastrointestinal and genitourinary bleeding) events. We undertook additional a priori subgroup analyses focused on ITP and CVST and post hoc subgroup analyses for DVT and PE^43^. Read codes (version 2) were used to determine adverse incident events recorded in the primary care electronic health record (Supplementary Table 6), which were then followed up in the linked RAPID dataset and National Records Scotland for hospitalization and mortality outcomes, respectively (Extended Data Fig. 4).

### Covariates

Data on the number of comorbidities recorded in the general practice record were derived from the QCovid risk groups on 1 December 2020 (ref. ^22^). Socioeconomic status was measured by quintiles of the Scottish Index of Multiple Deprivation^18^. The number of pre-vaccination program RT–PCR tests was extracted from the records as a marker of being in a population at high risk of exposure (for example, health and social care workers in whom regular testing was recommended).

### Statistical analysis

Conditional logistic regression analysis was used with case and control matching groups as the strata. The unadjusted model included no covariates other than the strata and the exposure. Separately, a simple adjustment was made for the number of clinical risk groups per patient^44^. Then, a full adjustment included socioeconomic status^24^ and number of RT–PCR tests an individual had before 8 December 2020, because we considered a priori these variables to affect vaccination receipt (all three) and risk of outcomes (risk groups and deprivation). Odds ratios for being a case among vaccinated versus unvaccinated individuals were estimated from the logistic regression, which, given the incidence–density sampling design, are mathematically equivalent to RRs. Unadjusted and adjusted RRs, along with 95% CIs, were calculated. Additionally, we investigated the individual QCovid risk groups as potential confounding variables (through statistical adjustment) or effect-modifying variables (through interaction)^44^. We also identified predictors (QCovid risk groups and age) for our combined outcomes events of interest, namely ITP and hemorrhage and arterial thromboembolic events.

We carried out an analysis of observed incident cases in the post-vaccination period compared to those expected from a pre-vaccination period. The pre-vaccination period used was the 28-d period after 1 October 2020. For the event to be incident, there were no thrombocytopenic, venous thromboembolic or hemorrhagic events in the same individual in the period from 1 September 2019. In the post-vaccination period, clinical events were linked to the vaccination records, and incident cases after vaccination were counted for the observed duration of the post-vaccination period. The expected events were the number of events per day in the pre-vaccination period divided by the population and multiplied by the days at risk in the post-vaccination period for all vaccinated and then summed. The difference between expected and observed events during the post-vaccination period was used to calculate expected additional cases per 100,000 vaccine doses. CIs were obtained from a parametric bootstrap based on the Poisson distribution using 10,000 samples.

Analyses were carried out by one statistician (C.R.) and independently checked by additional statisticians (E.M., U.A. and R.M.). All analyses were carried out with R software version 3.6.1 (ref. ^45^).

### Post-hoc analyses

The nested case–control method was used in our primary analysis, as opposed to an SCCS method, because recurrent adverse events of interest were considered unlikely to be independent. Nonetheless, we investigated confounding by indication and carried out a post hoc SCSS analysis to estimate the risk of COVID-19 vaccination and the adverse events of interest using conditional logistic regression with an offset for the length of the risk period^39^. This analysis compared risk for the same individuals in the time periods before and after vaccination. We considered the time period from the date of first vaccination dose to 28 d after as the risk (exposed) period. The control (pre-risk) period was the 90-d period before 14 d before vaccination (that is, 15–104 d before vaccine receipt), allowing for a 14-d clearance period. The main comparisons were the rate of adverse events between (1) the risk period and the pre-risk period and (2) the clearance period and the pre-risk period (Fig. 1).

As an additional post hoc analysis to understand whether cases of ITP could have represented reactivation of disease and whether relevant prescriptions that could cause thrombocytopenia were prescribed and ITP-directed therapy after vaccination was administered, platelet counts for individuals with the outcome were extracted from the general practice electronic health record (Extended Data Fig. 4), and prescriptions were extracted that could cause thrombocytopenia (including amiodarone cephalosporins, ciprofloxacin, ethambutol, furosemide, glycoprotein IIb/IIIa inhibitors (tirofiban, abciximab and eptifibatide), haloperidol, heparin, ibuprofen, irinotecan, linezolid, mirtazapine, naproxen, oxaliplatin, paracetamol, penicillins, phenytoin, quinidine, quinine, rifampicin, salmeterol, sodium valproate, sulphonamides, tacrolimus thiazides, trimethoprim and vancomycin). The following ITP-related prescriptions were extracted: azathioprine, ciclosporin, cyclophosphamide, danazol, dapsone, intravenous immunoglobulin, mycophenolate, oral corticosteroids, rituximab, vinca alkaloids, eltrombopag and romiplostim therapy.

### Sensitivity analyses

To reduce the potential for ascertainment bias, we carried out a sensitivity analysis with a censoring date before the first media reports of possible thrombotic events associated with ChAdOx1 (that is, 21 February 2021)^46^. An analysis to explore any influence on testing (RT–PCR) positive before an event (restricting our analysis to those who never tested positive previously) was undertaken.

We carried out an SCCS analysis including unvaccinated individuals with an additional temporal stratification of calendar time to allow for any potential secular trends in the outcome. In this analysis, all events in the period from 26 August 2020 (the date 90 + 14 d before the start of vaccination) were considered. Individuals were divided into at-risk versus control periods—that is, those who were never vaccinated and vaccinated individuals <14 d before vaccination were labeled as unvaccinated. Vaccinated individuals could also be in the before-vaccination clearance period or post-vaccination risk period. For this analysis, the incidence of events in the vaccine-specific risk period was compared to that in the unvaccinated.

### Reporting

To minimize the risks of individuals being identified, well-established safeguards were in place granting data access only to a limited number of accredited investigators, limiting access to identifiable personal information and mandating statistical disclosure control policies. For instance, we are unable to report results based on five events or fewer or undertake specific case reviews of individuals’ data^30^. We followed the Reporting of Studies Conducted using Observational Routinely Collected Data^47^ and Strengthening the Reporting of Observational Studies in Epidemiology^48^ checklists to guide transparent reporting of this cohort study (Supplementary Table 7).

### Reporting Summary

Further information on research design is available in the Nature Research Reporting Summary linked to this article.

## Online content

Any methods, additional references, Nature Research reporting summaries, source data, extended data, supplementary information, acknowledgements, peer review information; details of author contributions and competing interests; and statements of data and code availability are available at 10.1038/s41591-021-01408-4.

## Supplementary information

Supplementary Information Supplementary Tables 1–7

Reporting Summary

## Data Availability

A data dictionary covering the datasets used in this study can be found at https://github.com/ EAVE-II/EAVE-II- data-dictionary. The data that support the findings of this study are not publicly available because they are based on de-identified national clinical records. These are, however, available by application via Scotland’s National Safe Haven from Public Health Scotland. The data used in this study can be accessed by researchers through NHS Scotland’s Public Benefit and Privacy Panel via its Electronic Data Research and Innovation Service^49^.

## Code availability

All code used in this study is publicly available at https://github.com/ EAVE-II/Covid- vaccine-safety-haemo.
