# Supplementary material for: First-dose ChAdOx1 and BNT162b2 COVID-19 vaccines and thrombocytopenic, thromboembolic and hemorrhagic events in Scotland
Source: Nat Med. 2021 Jun 9;27(7):1290–7. doi: 10.1038/s41591-021-01408-4 (PMC8282499; doi:10.1038/s41591-021-01408-4)
Supplement: Supplementary file 2 — Reporting Summary [file 41591_2021_1408_MOESM2_ESM.pdf]

nature research

Corresponding author(s): Aziz Sheikh

Last updated by author(s): May 25, 2021

Reporting Summary

Nature Research wishes to improve the reproducibility of the work that we publish. This form provides structure for consistency and transparency in reporting. For further information on Nature Research policies, see our Editorial Policies and the Editorial Policy Checklist.

Statistics

For all statistical analyses, confirm that the following items are present in the figure legend, table legend, main text, or Methods section.

n/a	Confirmed	
☐	☒	The exact sample size (*n*) for each experimental group/condition, given as a discrete number and unit of measurement
☐	☒	A statement on whether measurements were taken from distinct samples or whether the same sample was measured repeatedly
☐	☒	The statistical test(s) used AND whether they are one- or two-sided<br>*Only common tests should be described solely by name; describe more complex techniques in the Methods section.*
☐	☒	A description of all covariates tested
☐	☒	A description of any assumptions or corrections, such as tests of normality and adjustment for multiple comparisons
☐	☒	A full description of the statistical parameters including central tendency (e.g. means) or other basic estimates (e.g. regression coefficient) AND variation (e.g. standard deviation) or associated estimates of uncertainty (e.g. confidence intervals)
☐	☒	For null hypothesis testing, the test statistic (e.g. *F*, *t*, *r*) with confidence intervals, effect sizes, degrees of freedom and *P* value noted<br>*Give P values as exact values whenever suitable.*
☒	☐	For Bayesian analysis, information on the choice of priors and Markov chain Monte Carlo settings
☒	☐	For hierarchical and complex designs, identification of the appropriate level for tests and full reporting of outcomes
☐	☒	Estimates of effect sizes (e.g. Cohen's *d*, Pearson's *r*), indicating how they were calculated

Our web collection on statistics for biologists contains articles on many of the points above.

Software and code

Policy information about availability of computer code

Data collection	We used the Community Health Index (CHI) number to deterministically link all datasets with vaccination records in Public Health Scotland (PHS). Read codes (Version 2) were used to determine adverse incident events recorded in the primary care electronic health record (Supplementary Table S6), which were then followed up in the linked RAPID dataset and National Records Scotland for hospitalisation and mortality outcomes, respectively (Extended Data Figure 4).
Data analysis	All code used in this study is publicly available at https://github.com/EAVE-II/Covid-vaccine-safety-haemo. All analyses were carried out with R software, version 3.6.1.

For manuscripts utilizing custom algorithms or software that are central to the research but not yet described in published literature, software must be made available to editors and reviewers. We strongly encourage code deposition in a community repository (e.g. GitHub). See the Nature Research guidelines for submitting code & software for further information.

Data

Policy information about availability of data

All manuscripts must include a data availability statement. This statement should provide the following information, where applicable:

- Accession codes, unique identifiers, or web links for publicly available datasets
- A list of figures that have associated raw data
- A description of any restrictions on data availability

The National Health Service in Scotland (NHS Scotland) provides comprehensive health services that are free at the point-of-care for all residents. Our base population for this study was 5.4 million residents (~99% of the population) registered with a general medical practice (GP) in Scotland. Almost all residents in Scotland are registered with a GP and have a unique Community Health Index (CHI) number used by NHS Scotland. We used the CHI number to deterministically link all datasets with vaccination records in Public Health Scotland (PHS) (Extended Data Figure 4). Vaccination information was extracted from the GP records and the

Turas Vaccination Management Tool (TVMT) system; together these captured all vaccination records, including those vaccinated in general practices, community vaccination hubs and other settings such as care homes and hospitals in Scotland. Further details on the data sources used in this study are available in a published project protocol.

Field-specific reporting

Please select the one below that is the best fit for your research. If you are not sure, read the appropriate sections before making your selection.

☒ Life sciences ☐ Behavioural & social sciences ☐ Ecological, evolutionary & environmental sciences

For a reference copy of the document with all sections, see nature.com/documents/nr-reporting-summary-flat.pdf

Life sciences study design

All studies must disclose on these points even when the disclosure is negative.

Sample size	All Scotland population (>99%) analysis.
Data exclusions	Exclusion criteria: Aged 17 or less at the index date.
Replication	Replication was successful. All code used in this study is publicly available at https://github.com/EAVE-II/Covid-vaccine-safety-haemo.
Randomization	Randomization is not relevant to our study as this is an observational study using linked Scottish national data.
Blinding	Blinding is not relevant to our study as this is an observational study using linked Scottish national data.

Reporting for specific materials, systems and methods

We require information from authors about some types of materials, experimental systems and methods used in many studies. Here, indicate whether each material, system or method listed is relevant to your study. If you are not sure if a list item applies to your research, read the appropriate section before selecting a response.

Materials & experimental systems

n/a	Involved in the study	
☒	☐	Antibodies
☒	☐	Eukaryotic cell lines
☒	☐	Palaeontology and archaeology
☒	☐	Animals and other organisms
☒	☐	Human research participants
☒	☐	Clinical data
☒	☐	Dual use research of concern

Methods

n/a	Involved in the study	
☒	☐	ChIP-seq
☒	☐	Flow cytometry
☒	☐	MRI-based neuroimaging
